# An “Uncommon” Role for Cyclic Enterobacterial Common Antigen in Maintaining Outer Membrane Integrity

**DOI:** 10.1128/mBio.02162-18

**Published:** 2018-10-30

**Authors:** Corey S. Westfall

**Affiliations:** a Washington University in St. Louis, St. Louis, Missouri, USA

**Keywords:** ECA, enterobacterial common antigen, *Escherichia coli*, outer membrane

## Abstract

Although discovered over 50 years ago, the physiological role of enterobacterial common antigen, a surface antigen produced by all members of the *Enterobacteriaceae*, has been poorly understood. In the work of Mitchell et al. (mBio 9:e01321-18, 2018, https://doi.org/10.1128/mBio.01321-18), the cyclized version of enterobacterial common antigen has been shown to play a role in maintaining the outer membrane permeability barrier, possibly through the inner membrane protein YhdP. This work also provides the tests needed to separate true effects from the numerous possible artifacts possible with mutations in enterobacterial common antigen synthesis.

## Abstract (precis)

Although discovered over 50 years ago, the physiological role of enterobacterial common antigen, a surface antigen produced by all members of the *Enterobacteriaceae*, has been poorly understood. In the work of Mitchell et al. (mBio 9:e01321-18, 2018, https://doi.org/10.1128/mBio.01321-18), the cyclized version of enterobacterial common antigen has been shown to play a role in maintaining the outer membrane permeability barrier, possibly through the inner membrane protein YhdP.

## COMMENTARY

The outer membrane (OM) of Gram-negative bacteria serves as a key permeability barrier, insulating the cell from toxic molecules, including bile salts, detergents, and some antibiotics (reviewed in reference 1). While the principal component of the outer leaflet of the OM is lipopolysaccharide (LPS), in *Enterobacteriaceae*, the OM outer leaflet also contains a carbohydrate-derived molecule of elusive function, enterobacterial common antigen (ECA) (2). Discovered as a cross-reacting antigen in Escherichia coli in 1962 (2), ECA consists of repeating units of the sugars *N*-acetylglucosamine, *N*-acetyl-d-mannosaminuronic acid, and 4-acetamido-4,6-dideoxy-d-galactose (reviewed in reference 3).

Historically, attempts to understand the physiological role of ECA have been confounded by several factors. Notably, ECA, O-antigen, and peptidoglycan biosynthesis all share a precursor pool; thus, impairments in one pathway often indirectly affect others, principally through modulating availability of the common precursor undecaprenyl-phosphate (UPP) (4). Further complicating study, ECA exists in three forms, each of which share a biosynthetic pathway. In the OM, ECA can be covalently linked to either LPS or to the lipid phosphatidylglyceride. ECA can also be found in the periplasm in a non-lipid-conjugated, cyclic form (ECA_cyc_). These chemical modifications are reviewed in reference 3. Difficulty in genetically separating these compounds, as well as challenges measuring their abundance, has impeded understanding of the specific contributions of each form to bacterial physiology. Finally, as a result of the interconnection between ECA, O-antigen, and peptidoglycan biosynthesis, mutations in ECA synthesis genes in E. coli and other *Enterobacteriaceae* often lead to induction of cell envelope stress pathways, making it difficult to deconvolute direct and indirect effects of genetic perturbations (4, 5).

New work from Mitchell et al. (6) circumvents these challenges to demonstrate that ECA_cyc_ plays a specific role in maintaining the permeability barrier and integrity of E. coli’s OM. Notably, the authors did not begin by investigating ECA_cyc_ directly but instead were led to this molecule through their work on a gene of unknown function, *yhdP*. Identified in a screen for mutants sensitive to SDS treatment in stationary phase, *yhdP* mutants are also sensitive to the glycopeptide antibiotic vancomycin, indicative of an increase in the OM permeability. The authors then performed a secondary screen for suppressors that restored both the vancomycin and SDS resistance, all of which mapped to the ECA biosynthetic operon (the *wec* operon). Further analysis established perturbations in ECA synthesis as the primary factor underlying suppression, independent of other cell envelope stress response pathways and UPP availability.

Armed with the knowledge that ECA biosynthesis and YhdP are intimately linked, the authors next determined the role of each of the ECA variants in Δ*yhdP* suppression. By carefully analyzing mutants at different steps of ECA synthesis, they were able to rule out loss of the lipopolysaccharide-attached ECA as contributing to suppression of the Δ*yhdP* phenotype, leaving the OM-associated phosphatidylglyceride-linked ECA and the periplasmic ECA_cyc_ as candidate effectors. Although differentiating between these two forms is complicated by the fact that the gene responsible for ECA_cyc_ synthesis (*wzzE*) is also responsible for regulation of ECA chain length, the authors cleverly used changes in temperature (an environmental determinant in ECA chain length) to rule out changes in ECA chain length as the cause of *yhdP* mutant OM permeability suppression. This firmly establishes that loss of ECA_cyc_ specifically is responsible for this phenotype.

**FIG 1:**
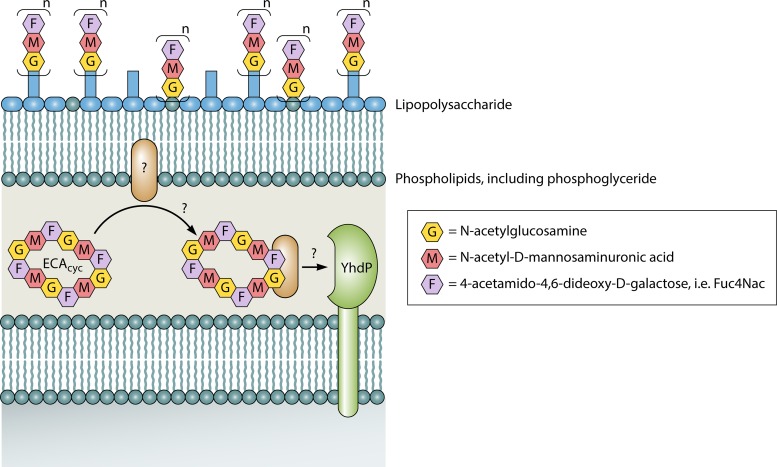
A proposed model for ECA_cyc_ in maintaining outer membrane stability. ECA_cyc_ could possibly bind and remove certain molecules from the outer membrane and target them to YhdP.

Together, this work illustrates a central role of ECA_cyc_ and YhdP in OM integrity, in which ECA_cyc_ serves a role in maintaining the OM permeability barrier and YhdP controls the activity of ECA_cyc_. YhdP regulation appears to be important as the authors found that the *yhdP* gene cooccurs with ECA synthetic genes in the *Enterobacteriaceae*. Interestingly, YhdP is predicted to localize to the inner membrane where it could directly interact with ECA_cyc_, but low homology to the poorly characterized AsmA family of proteins to which *yhdP* belongs prevents easy interpretation of function. Although future work is required to understand the nature of this interaction along with the mechanism connecting ECA_cyc_ to OM integrity, the authors posit that ECA_cyc_ may be involved in transfer of specific molecules from the periplasm to the OM or vice versa, similar to the activity of some cyclodextrins that can pull cholesterol out of membranes without disrupting the membrane. In this model (Fig. 1), either YhdP could directly interact with ECA_cyc_, perhaps regulating its activity or even potentially even acting as an ECA_cyc_ receptor, or ECA_cyc_ could take target molecules from the OM and transfer them to YhdP. Whether through this or an alternative mechanism, the work by Mitchell et al. has opened up a fascinating new field of study on the impact of this so-called “common” molecule and has provided the first insight into the physiological role of at least one form of ECA.
